# Automatic Stem Mapping by Merging Several Terrestrial Laser Scans at the Feature and Decision Levels

**DOI:** 10.3390/s130201614

**Published:** 2013-01-25

**Authors:** Xinlian Liang, Juha Hyyppä

**Affiliations:** Department of Remote Sensing and Photogrammetry, Finnish Geodetic Institute, FI-02431 Masala, Finland; E-Mail: juha.hyyppa@fgi.fi

**Keywords:** forestry, terrestrial laser scanning, LiDAR, point cloud, single-scan, multi-single-scan, multi-scan, registration

## Abstract

Detailed up-to-date ground reference data have become increasingly important in quantitative forest inventories. Field reference data are conventionally collected at the sample plot level by means of manual measurements, which are both labor-intensive and time-consuming. In addition, the number of attributes collected from the tree stem is limited. More recently, terrestrial laser scanning (TLS), using both single-scan and multi-scan techniques, has proven to be a promising solution for efficient stem mapping at the plot level. In the single-scan method, the laser scanner is placed at the center of the plot, creating only one scan, and all trees are mapped from the single-scan point cloud. Consequently, the occlusion of stems increases as the range of the scanner increases, depending on the forest's attributes. In the conventional multi-scan method, several scans are made simultaneously inside and outside of the plot to collect point clouds representing all trees within the plot, and these scans are accurately co-registered by using artificial reference targets manually placed throughout the plot. The additional difficulty of applying the multi-scan method is due to the point-cloud registration of several scans not being fully automated yet. This paper proposes a multi-single-scan (MSS) method to map the sample plot. The method does not require artificial reference targets placed on the plot or point-level registration. The MSS method is based on the fully automated processing of each scan independently and on the merging of the stem positions automatically detected from multiple scans to accurately map the sample plot. The proposed MSS method was tested on five dense forest plots. The results show that the MSS method significantly improves the stem-detection accuracy compared with the single-scan approach and achieves a mapping accuracy similar to that achieved with the multi-scan method, without the need for the point-level registration.

## 1. Introduction

The reference data collected from sample plots are fundamental parameters for forest-related studies. They are indispensable inputs to the biological, ecological, and geospatial modeling of standing trees for purposes such as the development of nationwide allometric models to estimate forest parameters [1], the calibration of estimation models developed from remote sensing techniques in national forest inventories [2] and the study of ecological characteristics [3].

Field reference data are conventionally collected at the sample plot level using manual measurements. The number of attributes collected from stems is limited. Calipers, measuring tapes and hypsometers are the most common measuring tools used for the retrieval of stem attributes. They are low-cost and relatively reliable, but labor-intensive [4,5]. Some of the most important attributes (e.g., the stem curve of standing trees) are not measurable by these tools. A conventional field inventory relies on the Diameter at Breast Height (DBH), possibly the diameter at a height of 6 m, and on the tree height. More automated and more cost-effective techniques are needed to provide field inventory data.

More recently, terrestrial laser scanning (TLS), also known as ground-based LiDAR, has been demonstrated to be a promising solution for obtaining field inventory data at the sample plot level (e.g., [6–10]). The data collected using TLS form a three-dimensional (3-D) point cloud that documents the forest horizontal and vertical structures. The main advantage of using TLS in forest field inventories lies in its capacity to document the forest in detail. Under optimal conditions (e.g., in the leaf-off season) and in the application of sophisticated processing methods, TLS can provide both accurate and cost-effective stand attributes for forestry applications. TLS data also permit time series analyses because the entire plot can be consecutively documented over time [11]. At present, the application of TLS data in forest inventories is hampered by the challenges in the automation of point cloud processing. Other important factors hampering the use of the technology include the relatively high cost of the instrument, the shortage of software, and the lack of personnel training. Additionally, it should be noted that acceptable results, from the forester's perspective, obtained with TLS for plot-level inventory have only recently been presented, so it will take some time for foresters to start using TLS operationally.

Both single-scan and multi-scan methods can be employed in TLS measurement. In the single-scan approach, the laser scanner is placed at the center of the plot, and one full field-of-view (e.g., 360-degrees-by-310-degrees) scan is made. In the multi-scan method, several scans are made simultaneously inside and outside of the sample plot. The single-scan method takes less time to collect the field data than the multi-scan method. However, in the single-scan data, the objects behind the nearest surfaces in the direction of the laser beams are missed. Studies have shown that 10–32% of all trees in the sample plot are not scanned from the plot center because they are occluded by other trees closer to the scanning position [12–15]. The occlusion effect increases with increasing distance from the scanner. To be able to cover almost 100% of the trees, the multi-scan method is necessary. The multi-scan method is assumed to be the most accurate technique for mapping a sample plot, but it is not always practical due to the cost of the manual or semi-automated processing needed in the registration of several scans.

The registration of several scans covering the study area is a crucial step in the interpretation of multi-scan data. At the moment, fully automated registration between several scans at the point level is challenging.

The matching of several scans can be achieved using different methods. For example, in the built environment, flat reflectors are commonly used as reference targets. High matching accuracy (e.g., at the millimeter level) can be achieved. The scans can also be matched to each other or to the mapping coordinate system with the help of the total station, which accurately measures scanning locations.

The registration of several scans on the forest plot is currently accomplished by applying artificial objects as the reference targets, and the co-registration is performed manually or semi-automatically using these targets. Spheres are commonly employed because the determination of the ball center is not significantly influenced by the scanning angle. However, the use of artificial reference targets requires additional effort and knowledge. The field crews needs to carry those reference targets and tripods throughout the field measurement, and they need to have the experience to use them properly (e.g., to ensure the targets have good network geometry). An automated solution that does not use artificial reference targets would clearly increase the efficiency of the field measurements and the post-processing, which is the aim of this paper.

The registration of several individual scans is often referred to as the match of scans at the point-level. In fact, merging or fusion can be performed at different levels, according to the stage at which the registration is performed. Point-level registration transforms several point clouds into a common reference system, and the interpretation of the sample plot is made in the merged data set. At the feature level, features (e.g., tree attributes) extracted from different scans are matched to each other and integrated for further analyses. At the decision level, the individual data sets are processed separately, and the information obtained is combined to support the interpretation of the forest sample plot.

A multi-single-scan (MSS) method is proposed in this paper. The MSS method first processes several TLS scans individually and then combines the tree stem attributes obtained at the feature and decision levels to support the forest plot-level interpretation. The MSS method provides a new possibility for mapping sample plots without using artificial targets and point-level registration. Thus, the field data collection of the MSS method is less demanding.

## 2. Study Material

### 2.1. Study Area and Field Reference

The study area is a managed forest located in the vicinity of Evo, Finland (61.19°N, 25.11°E). The self-build method was tested on five circular plots, each with a fixed radius of 10 meters. The main tree species growing on the plots considered in this study are Scots pine (*Pinus sylvestris*), Norway spruce (*Picea abies*), and birch (*Betula sp.*). Two of the plots are located in a mixed forest. Pine and spruce account for approximately 75% of the trees on each plot, and deciduous species account for approximately 25%. Two of the plots are pine-dominated, and one plot is spruce-dominated. The densities of the study plots are between 605 and 1,210 stems/ha; thus, these plots were categorized as high-density plots based on the threshold number of 600 stems/ha, as in [16].

The trees on the study plots are at various stages of growth. Both small and dominant trees are present. The DBH ranges from 6 cm to 41 cm, and the tree heights range from 4 m to 27 m. The standard deviation (std) of the DBH varies between 3.3 cm and 8.7 cm at the plot level. The std of the tree height varied between 1.8 m and 7.3 m. Descriptive statistics of the plots at the time of the field inventory are summarized in Table 1.

Field references were collected between 2007 and 2009. The coordinates of the plot centers were measured using a Trimble GEOXM 2005 GPS device and post-processed using local base station data. Trees were located using a Suunto bearing compass (Suunto Oy, Vantaa, Finland) and a Haglöf Vertex laser rangefinder (Haglöf Sweden AB, Långsele, Sweden). The locations of the trees were calculated using angle, distance, and plot center data. The absolute spatial accuracies of the stem location measurements vary depending on the plot attributes. The relative accuracy should be good because each stem location is referenced to the same point, *i.e.*, the plot center. The DBH was measured to the nearest millimeter using calipers. All trees with a DBH greater than 5 cm were included in the reference measurements.

### 2.2. Terrestrial Laser Data Acquisition

**Figure 1.**
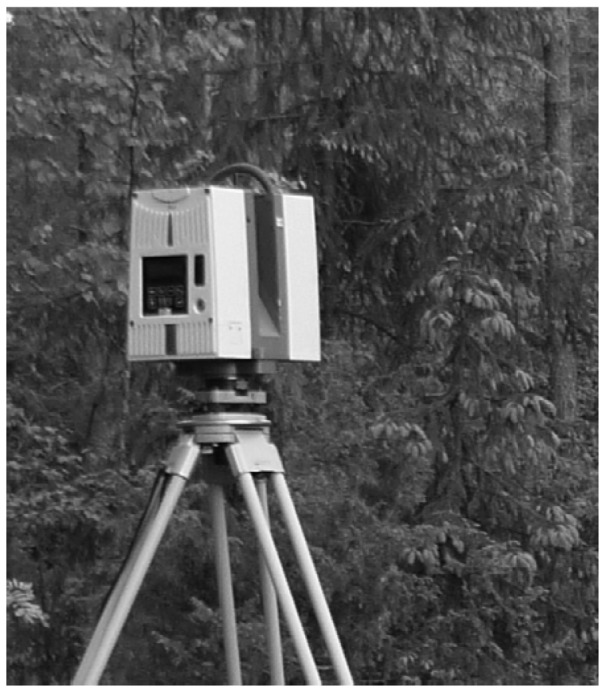
The terrestrial laser scanner.

The TLS data were collected in May 2010. A Leica HDS6100 terrestrial laser scanner (Leica Geosystems AG, Heerbrugg, Switzerland) was used in the data acquisition. The scanner employs a continuous wave of 650–690 nm to measure distances. The distance measurement accuracy is 2–5 mm. The maximum data acquisition rate is 508,000 points per second. Figure 1 shows the Leica HDS6100 laser scanner. Table 2 presents the detailed specifications of the scanner.

**Figure 2.**
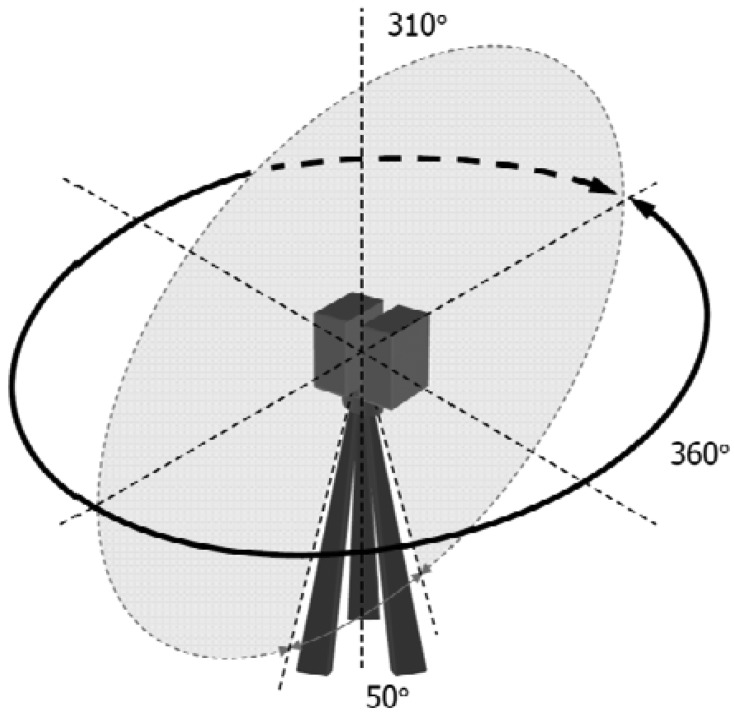
The scanning mechanism of the TLS scanner.

The scanner measures the surrounding environment stepwise in the horizontal and vertical directions, with a fast vertical mirror rotation and a slower horizontal instrument rotation. A full field-of-view scan is 360 degrees by 310 degrees. The scanning mechanism is illustrated in Figure 2.

**Figure 3.**
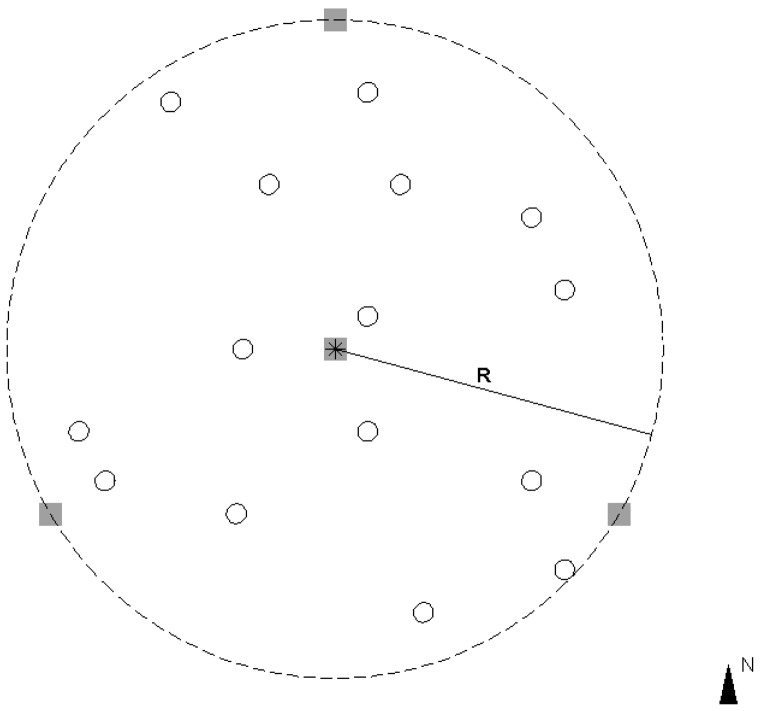
The scanning scenario. The sample plot is the circular area with a radius R. The plot center is marked by the asterisk, and the positions of the trees are shown as solid circles. The gray squares indicate the scanning locations.

The plots were scanned without any pre-scan preparations (e.g., the removal of lower tree branches or the clearance of undergrowth). Four scans inside and outside of each plot were used in this study. Figure 3 illustrates the scanning scenario. One scan was performed at the plot center, and the other three scans were conducted at the plot border. The scanning positions at the border were approximately north, southeast, and southwest of each plot.

A full field-of-view scan was performed at each scanning position. The scanning resolution was set at an angular increment of 0.036° in both the horizontal and vertical directions. The point distance is 6.3 mm at a distance of 10 meters, measured between the beam centers on a surface perpendicular to the laser beam.

## 3. Methods

**Figure 4.**
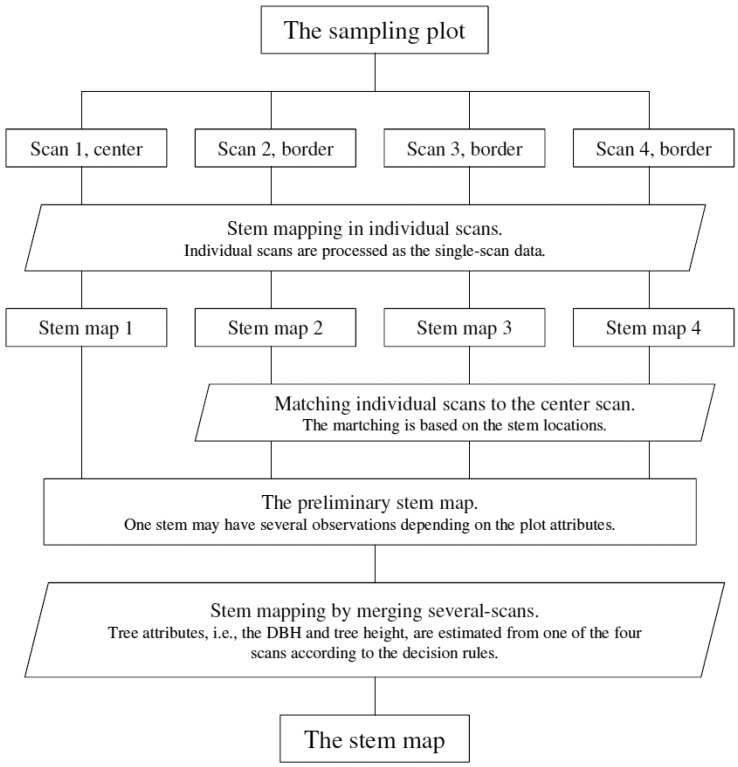
Conceptual diagram of the MSS method.

The MSS method maps the sample plot in individual scans first and then merges the results to map the sample plot, as illustrated in Figure 4. Tree attributes (*i.e.*, stem locations) are extracted from different scans and matched to each other at the feature level. The information obtained from different scans, e.g., DBH, is combined at the decision level to support the interpretation of the sample plot.

**Figure 5.**
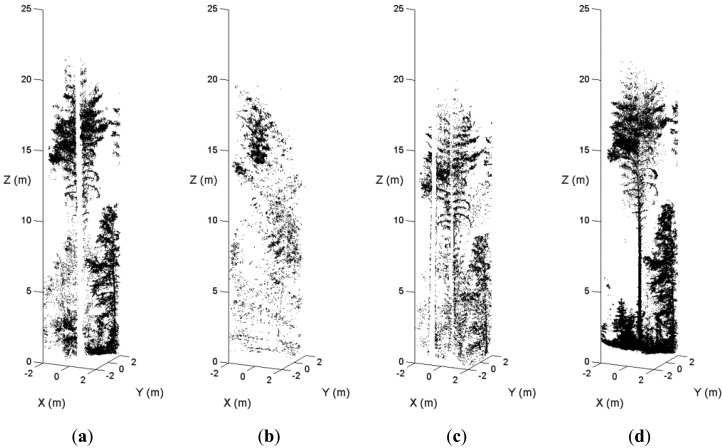
An example of the occlusion effect on a sample plot. The point clouds of a pine tree from the center scan and three border scans are shown in (**a**), (**b**), (**c**), and (**d**).

In dense forest plots, some tree stems are not recorded in the single-scan data because of shadowing. Figure 5 illustrates an example of the occlusion effect. A sample plot was scanned from the plot center and three scanning positions on the border. Figure 5(a) is the point cloud of a pine tree in the center scan. Figure 5(b,c,d) are the point clouds of the tree in the border scans. The stem of this pine tree was totally occluded by other trees in the center scan and two border scans. In the fourth scan, this tree is visible. The MSS method maps the tree in the point cloud where the visibility of the tree stem is good. The occlusion effect is minimized by merging scans at the feature and decision levels.

### 3.1. Stem Mapping in Individual Scans

Tree stems were automatically recognized and reconstructed using a robust modeling procedure. The stem locations in each scan were extracted as features for the subsequent matching process.

The original point cloud of the sample plot includes points reflected from the ground, stem, crown, and other objects in the plot. The stem points were automatically identified based on the spatial properties of the points. A local coordinate system was established for each point in its neighborhood. In this local coordinate system, the axis directions were defined by eigenvectors, and the variances of the data along the axes were indicated by eigenvalues. A point was on a surface if its neighboring points were distributed mainly along two axes in the local coordinate system. This point was most likely reflected from a tree stem if the direction of the normal vector to the surface was horizontal in the real world coordinate system.

**Figure 6.**
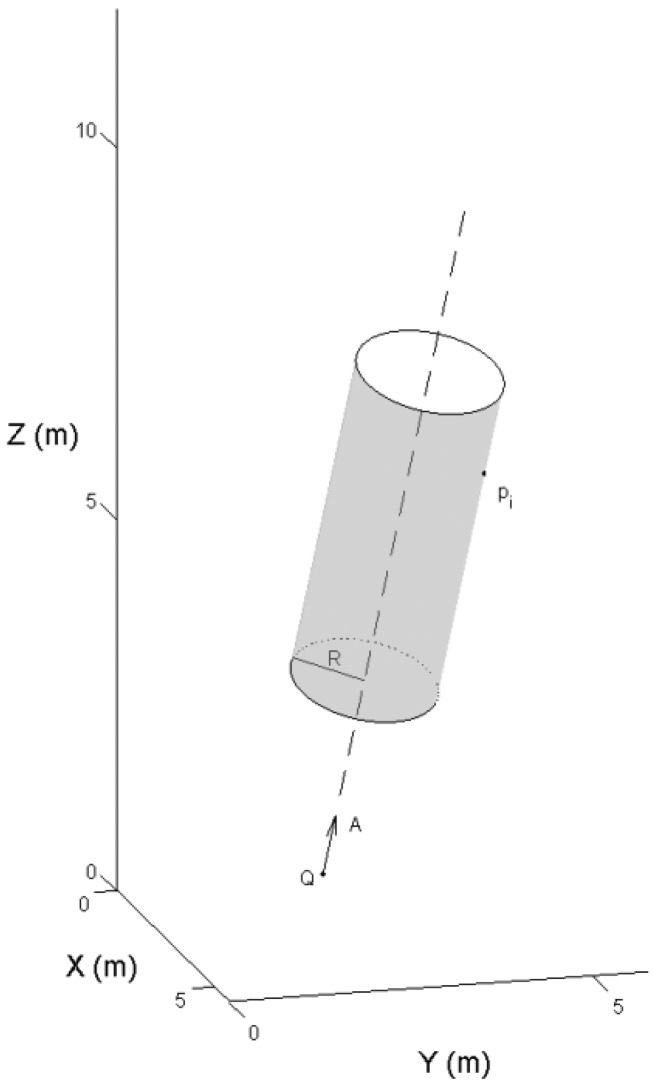
A cylinder and a point on its surface.

The stem model was reconstructed from the selected points. A tree stem was divided into a series of small sections along the stem profile. In each section, a 3-D cylinder was fit to the point cloud. The cylinder definition is given by Equation (1):

(1) ‖(P−Q)×A‖−R=0

where *P* = {*p_i_*, *i* = 1,2, …, *n*} indicates the points on the cylinder surface, *Q* = (*x_q_*, *y_q_*, *z_q_*)*^T^* is a point on the axis, *A* = (*a_x_*, *a_y_*, *a_z_*)*^T^* is the direction of the axis of the cylinder with a unit length, and *R* is the radius. Figure 6 illustrates a cylinder and a point on the cylinder surface.

**Figure 7.**
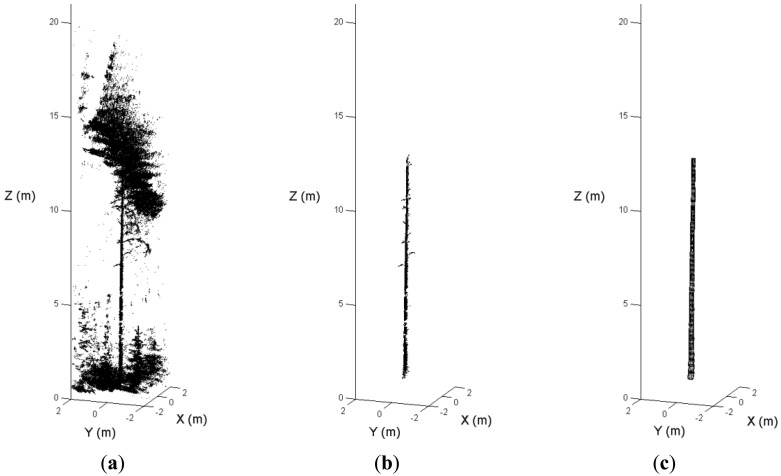
The automated stem reconstruction: (**a**) the original point clouds of a tree; (**b**) the detected stem points; and (**c**) the scan model automatically reconstructed.

The distance, or residual, between the selected laser point and the cylinder surface is given by ‖(*P* − *Q*) × *A*‖ − *R*. To eliminate the outliers from the branches and crowns, a weight was given to each point depending on the residual. More details on the robust modeling procedure were given in [15]. Figure 7 illustrates the steps of the automated stem reconstruction.

The model element at the breast height was selected. The diameter and position of the cylinder were employed as the estimation of the DBH and the stem location, respectively. The breast height was defined as a point 1.3 m above the ground level, and the ground height was estimated based on the lowest data point around the stem model. The tree height was calculated as the height difference between the highest laser point around the model and the ground level.

### 3.2. Matching Individual Scans

The individual scans were co-registered at the feature level using location-based matching. The border scans were matched to the center scan in two dimensions (2-D) based on the stem locations. The unknown parameters are the translations (X_0_, Y_0_) and rotation θ between two data sets.

Given two data sets, P and Q, with P ⊂ R^2^ and Q ⊂ R^2^, each group contains a subset P′ and Q′ in which a one-to-one correspondence exists, as shown in Equations (2) and (3):

(2) $${\left[X_{p},Y_{p}\right]}^{T}={\left[X_{0},Y_{0}\right]}^{T}+M{\left[X_{q},Y_{q}\right]}^{T}$$

(3) $$M=\left[\begin{array}{cc} \cos\theta & -\sin\theta \\ \sin\theta & \cos\theta \end{array}\right]$$

where *P* and *Q* have *m* and *n* points, respectively; *P*′ = {*p*_1_, *p*_2_, …, *p_k_*}, *Q*′ = {*q*_1_, *q*_2_, …, *q_k_*} and *k* ∈ [2, min(*m*, *n*)]; and *pi* = [*X_p_i__*, *Y_p_i__*], *q_j_* = [*X_q_j__*, *Y__q_j_*], *i*, *j* ∈ [1, *k*].

The least squares technique can be used to determine the registration parameters, given two pairs of corresponding points and an initial estimation. However, in this case, there is no prior knowledge available about the translation and rotation. The unknown parameters were estimated using a clustering technique proposed in [17]. The disadvantage of this method is that the computation effort of searching becomes huge when the number of unknown parameters is more than 2. In this study, the computation is further simplified to speed the clustering process.

Equation (2) expresses the correspondence by points in two data sets. In fact, the correspondence can also be expressed by vectors. Given two pairs of corresponding points, (p_i_, q_j_) and (p_i′_, q_j′_), the relationship between vectors $\vec{{\text{p}}_{ı}{\text{p}}_{{ı}^{\prime }}}$ and $\vec{{\text{q}}_{\text{j}}{\text{q}}_{{\text{j}}^{\prime }}}$ can be expressed by Equation (4):

(4) $${\left[X_{i}-X_{i^{\prime }},Y_{i}-Y_{i^{\prime }}\right]}^{T}=M{\left[X_{j}-X_{j^{\prime }},Y_{j}-Y_{j^{\prime }}\right]}^{T}$$

Equation (4) shows that the corresponding vectors can be expressed by rotation θ only, where $\;\theta =(\theta_{\vec{{\text{p}}_{ı}{\text{p}}_{{ı}^{\prime }}}}-\theta_{\vec{{\text{q}}_{\text{j}}{\text{q}}_{{\text{j}}^{\prime }}}})$. Given the rotation and a pair of matched points, the translation (X_0_, Y_0_) is given by Equation (5):

(5) $${\left[X_{0},\;Y_{0}\right]}^{T}={\left[X_{i},Y_{i}\right]}^{T}-M{\left[X_{j},Y_{j}\right]}^{T}$$

Equations (4) and (5) indicate that the determination of three transformation parameters is equivalent to the determination of a pair of matched points and the rotation angle. The unknown transformation parameters were therefore estimated in three consecutive steps: (i) find the rotation θ and a pair of matched points, (ii) determine the translations (X_0_, Y_0_), and (iii) calculate three parameters by least squares matching.

The rotation θ is determined by finding a pair of matched vectors that have the same rotation angle. All possible matched pairs were tested because there is no reliable initial estimation. For a point p_i_ in P and a point q_j_ in Q, all possible matching vectors were selected in which two vectors are similar in length. The vector pairs with the same rotation angle were accumulated in a one-dimensional accumulator. The maximum possible number of matched points between P and Q is min(m, n) − t, in which t is the number of points in P that do not have correspondences in Q. The matching process continues until the maximum number in the accumulator is reached, *i.e.*, k pairs of matching points were found.

The translations (X_0_, Y_0_) were then computed using a matched pair and the rotation angle, as in Equation (5). The final transformation parameters and corresponding points were estimated by least squares matching method.

### 3.3. Stem Mapping by Merging Several-Scans

Individual stem maps were transformed into the coordinate system of the center scan using the estimated transformation parameters. The estimations of the tree location and DBH were selected from the scan in which the stem stood closest to the scanning position. The tree height estimations were selected, in contrast, from the scan in which the distance between the stem and scanning position was the greatest. The visibility of the lower part of the stem was assumed to be better if the stem stood closer to the scanning position. The possibility that a tree top is hit by a laser beam is assumed to be greater if the distance between the stem and the scanner is larger. The scanner was placed at the ground level. The heights of the trees were typically several tens of meters. Previous research has shown that tree height is typically underestimated. Additional discussion of the merging strategy is provided in Section 5.

### 3.4. Evaluation of the MSS Method

The results of the MSS method were compared with manual field measurements obtained at the plot level. The accuracy of the estimations was evaluated using the bias and root mean squared error (RMSE), as defined in Equations (6) and (7):

(6) $$\text{Bias}=\frac{1}{n}\sum_{i=1}^{n}e_{i}=\frac{1}{n}\sum_{i=1}^{n}(y_{i}-y_{ri})$$

(7) $$\text{RMSE}=\sqrt{\frac{\sum^{\;}{\left(y_{i}-y_{ri}\right)}^{2}}{n}}$$

where y_i_ is the ith estimation, y_ri_ is the ith reference, and n is the number of estimations. The relative RMSE was obtained by dividing the RMSE from Equation (7) by the mean of the reference values.

The results of the MSS method were also compared with the standard single-scan method to evaluate the relative performance of the MSS method.

## 4. Results

The results of the stem mapping using the MSS method are reported in Table 3. The stem-detection accuracy was between 92% and 100% at the plot level. The overall stem-detection accuracy was 95.3%. The RMSE of the DBH estimation ranged from 0.90 cm to 1.90 cm. The RMSE of the tree height estimation ranged from 2.04 m to 6.53 m.

For comparison, the five test plots were also mapped using the single-scan method. The center scan, which was also used in the MSS method, was processed in the same manner as the single-scan data. Table 4 summarizes the results of the stem mapping by the single-scan method. The stem detection rate varied between 67% and 90%. The overall detection rate was 73.4%, which is close to that reported in [15]. The RMSE of the DBH estimation ranged from 0.74 cm to 2.41 cm. The RMSE of the tree height estimation ranged from 1.36 m to 4.29 m.

**Figure 8.**
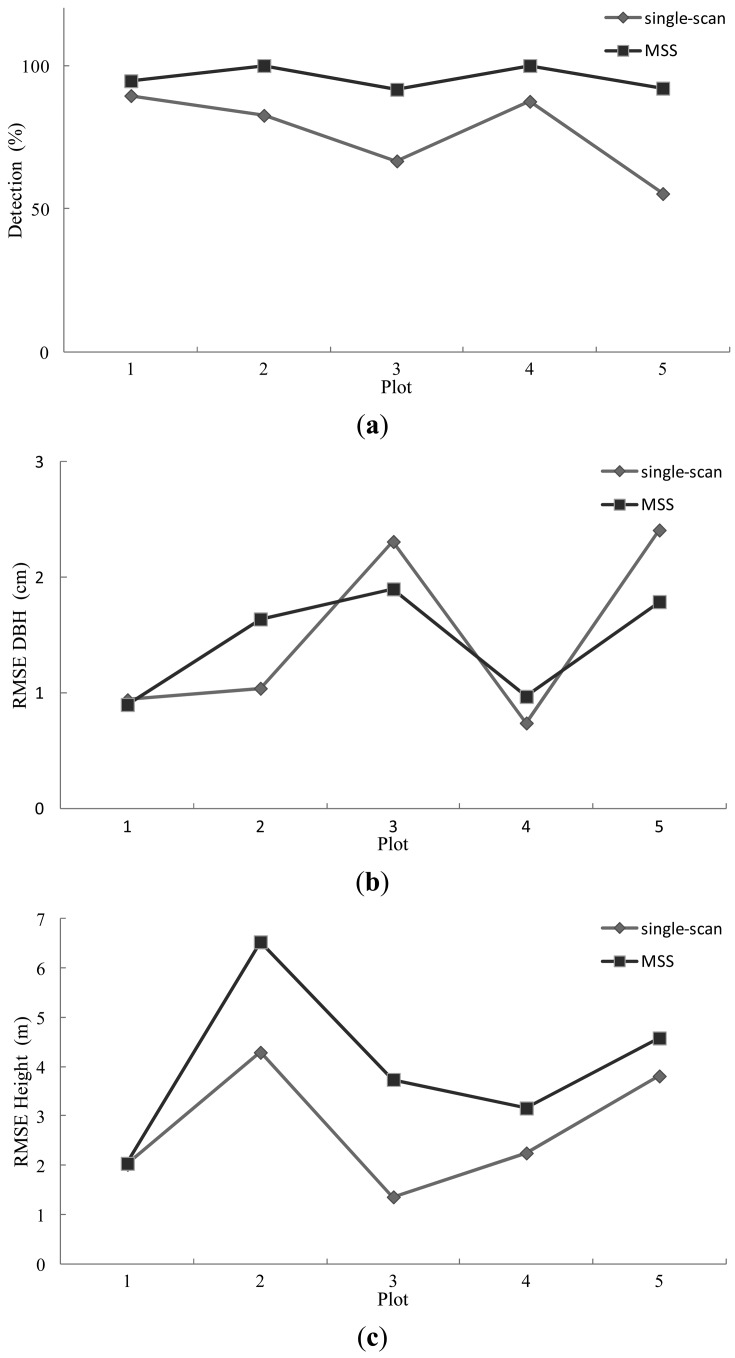
The mapping results using the MSS and single-scan methods: (**a**) the detection rate; (**b**) the RMSE of the DBH estimation; (**c**) the RMSE of the tree height estimation.

Figure 8 illustrates the mapping results using the MSS and single-scan methods. As shown in the figure, the MSS method improves the stem-detection accuracy significantly and the DBH estimation slightly. The tree height was likely overestimated by the MSS method.

Some reference stems were not detected in the point clouds by the MSS and single-scan methods. Table 5 summarizes the attributes of those stems.

## 5. Discussion

The MSS method achieved high stem-detection accuracy without using artificial targets to merge multiple scans. Therefore, the efficiency of field data measurement is clearly improved by using the MSS method.

In theory, the multi-scan approach is the best method for mapping a sample plot with respect to the estimation of the tree attributes, because trees are fully covered by the merged point cloud. The equipment used in the multi-scan method includes one scanner, several reference targets, and tripods for the scanner and every target. Six balls are typically used on a plot with a 10 m radius. The number of reference targets and tripods is determined by the plot size, tree attributes, and forest density. The field team must have at least two persons to carry and operate the required instruments. In the MSS method, the equipment includes only one scanner and its tripod. The measurement can be made by one field crew. The workload for the MSS method is clearly lower than that for the multi-scan method. Meanwhile, the MSS method also makes the movement between sample plots easier because fewer instruments are employed. Therefore, the number of sample plots that can be measured by the MSS method is expected to be greater than the number that can measured by the multi-scan method in the same amount of time. It is also possible to increase the number of sample plots measured by mounting TLS equipment in a moving vehicle, such as a skidoo or an all-terrain vehicle. This is now more feasible because no artificial reference targets are needed. Both stop-and-go and continuously moving (referring to mobile laser scanning) scenarios can be studied. The adaptation of a moving vehicle carrying TLS equipment and the use of MSS may significantly reduce the time needed to map a single forest plot.

Table 6 summarizes the mapping results of the multi-scan method as reported in earlier references. Three to five scans per plot have typically been employed with the multi-scan method, which is similar to the number used with the MSS method. The stem-detection accuracy using the multi-scan method has been reported as being between 93% and 100%. The mapping results presented in this paper, as reported in Table 3, show that the MSS method achieves a mapping accuracy on dense forest plots similar to that of the multi-scan method on sparse plots. In addition, the MSS method achieves this accuracy without point-level registration using reference targets, for which no automated solution yet exists.

The RMSE of the DBH estimation by the MSS method varied between 0.90 cm and 1.90 cm, which is better than most results reported so far, as shown in Table 6. The RMSE of the DBH estimation using the conventional multi-scan method in past studies was between 1.48 cm and 5.69 cm. In theory, the conventional multi-scan method should be superior with respect to the DBH estimation, suggesting that improvements can be made with previously reported multi-scan methods. The estimation accuracy depends on the tree attributes and the modeling method. For example, the DBH has been typically estimated in a horizontal 2-D plane in previous studies. This method assumes that the tree stem stands straight up. In practice, however, the stem section may lean in directions other than the vertical. The DBH measured in the 2-D plane is a rough estimation. In the present method, a stem section was estimated using a 3-D cylinder model and a robust fitting procedure. The direction and radius of the stem were estimated simultaneously.

The single-scan approach measures the sample plot much faster than the multi-scan method. Only one scan was made on the plot (e.g., at the plot center). The major problem with this method is the low detection rate. The MSS method significantly improved the stem-detection accuracy compared with the single-scan approach. In the experiment, there were 5.2%, 17.4%, 25%, 12.5%, and 36.8% more stems detected by the MSS method, as illustrated in Figure 9(a). The improvement is remarkable on plots where the stem density is high (e.g., more than 1,000 stems/ha), and it generally decreases as the density decreases.

The MSS method also slightly improves the DBH estimation, as shown in Figure 9(b). In the MSS method, the stem was mapped in the scan in which the stem was closest to the scanning position. This approach assumes that the stem is likely to be captured and accurately reconstructed if it is close to the scanner. The improved DBH estimation indicates that this approach works well. However, this assumption may not always be valid. The scanning geometry may significantly influence the visibility of the stem in the point cloud, especially within dense forest plots [15].

Tree height estimation using TLS at the plot level has not been studied much. Previous results showed that tree height is typically underestimated, and that the magnitude of estimation error is typically several meters. Huang *et al.* [22] reported a –0.26 m bias and a 0.76 m RMSE on one plot (212 stems/ha) by the multi-scan method. Brolly and Kiraly [12] reported a –0.27 m bias with a 1.82 m RMSE and a –2.37 m bias with a 3.25 m RMSE on one plot (753 stems/ha) with the single-scan method. Hopkinson *et al.* [9] reported an approximately 1.5 m underestimation of tree heights on two plots (465 and 661 stems/ha) by the multi-scan method. Maas *et al.* [20] reported a –0.64 m bias and a 4.55 m RMSE for 9 selected trees on four plots (212–410 stems/ha) by the single- and multi-scan methods. The typical reason for underestimation of tree height by TLS is the occlusion of tree tops due to shadowing by other parts of the crown, *i.e.*, wide crowns of tall trees do not allow a nearby scanner to see the tree tops.

Tree height was typically overestimated by the MSS method in this study. The RMSE for the height estimation was between 2.04 m and 6.53 m at plot level, implying that the results are not yet at an acceptable level for operative plot-level inventory. The overestimation of tree height is attributed to the stand structure, tree growth, accuracy of the reference data, and air points in the point cloud.

On the test plots, there are many young trees, and the plot density is high. The highest point around the stem position is not a good estimation of the tree top when a small tree stands next to bigger ones. The merging algorithm in the MSS method seemed sensitive to this phenomenon, and therefore false tree heights were obtained. The estimation of tree height with the single-scan method was better than with the MSS method. This is most likely because there are fewer points reflected from objects far from the scanner due to the occlusion effect, and therefore the overestimation is less significant with the single-scan method than with the MSS method.

Tree growth also leads to overestimation, especially with young trees. In this study, the TLS data were collected one to three years after the field reference measurement. It is possible for a young tree to grow 0.5 m to 1 m (and up to 2 m with saplings) within two years. The estimation should be more accurate if the field reference and TLS data were collected at the same time. Meanwhile, the error of the tree height estimation using TLS data includes the error of reference data. The accuracy of the conventional reference measurement using a hypsometer ranges from 0.5 m to 1.5 m, depending on the tree species (crown type), terrain slope, and forest density. This factor has also been mentioned in other studies (e.g., [20]). The fourth factor is the air points. In the point cloud, some points were not reflected from any real objects in the scene but were noise introduced by the intersection of the laser beam and object edges. The tree height is overestimated if there are such points around the stem location. The number of these points can be reduced by filtering during the preprocessing stage.

In the MSS and single-scan approaches, the same robust modeling method was employed in the mapping procedure. The attributes of the missed reference stems, as summarized in Table 5, show that both small and large trees may not be detected, but the missed reference stems were mainly small ones. This result indicates that the robust modeling method effectively detects stems in the point cloud. The MSS method significantly improves the detecting accuracy compared with the single-scan approach. This shows the scanning geometry, which leads to the shadow effect, clearly influences the mapping result. Several scans are necessary to map almost 100% of trees on the sample plot. Table 5 also shows that the stem mapping becomes more difficult as the distance between the stem and the scanner increases. The mean distance between the missed stem and the scanning position was more than 8 m when the single-scan method was employed. This is consistent with previous research. In [15], the detection accuracy was shown to decrease as a function of the range.

A new mapping method for the measurement of forest sample plots using several single-scans is reported in this paper. The MSS method achieves high mapping accuracy and reduces the cost of field data collection. In addition to these main benefits, the MSS method also provides a solution for several applications that should be further studied.

First, the MSS method does not rely on artificial reference targets to merge multiple scans, and therefore, the field reference can be measured over a large area. The permanent sample plot is typically a small area of the forest with a radius of approximately 10 m in the national forest inventories. In practice, a large sample plot is desirable. A large sample plot not only provides a more accurate and comprehensive understanding of the forest environment but also makes the registration of the ground reference and airborne remote sensing data easier. However, using conventional measurement methods to collect reference data on circular plots with radii larger than 10 to 12 m is very demanding and mostly impractical for forest inventories, especially if the stem count per hectare is high. The experiments in this paper showed that nearby scans can be automatically matched. By applying an idea similar to that proposed in the MSS method, nearby scans made within a large area could be matched and transformed into a common system to collect the field reference data.

Second, the MSS method uses stem locations as features to match several scans and should therefore be scalable to larger-size plots. A new application would be to map a large sample area using only a few scans. The unknown parameter is the practical size of the sample plot. The common trees between two neighboring scans account for approximately 50% of all trees in a scan (this percentage is explained later). The percentage of the common stems may decrease as the plot size increases, because the stem-detection accuracy decreases with increasing range [15]. Therefore, the matching of two data sets can be difficult, where half or more than half of the data in one set do not have correspondences in another data set. However, it is possible to match two data sets with large radii if the detecting accuracy is not decreased dramatically at the far end of the plot. Research has shown that trees far from the scanning position can be mapped. The radius of the sample plot was 30 m in the study by Brolly and Kiraly [12] and 50 m in the study by Lovell *et al.* [13]. In the study by Liang *et al.* [24], trees standing 50 m to 60 m away from the scanner were mapped. An interesting question is what the practical size of the sample plot is for mapping a large area using the MSS method. Further work is needed to explore this. The expense of field forest inventories would decrease dramatically if this idea is proven to be viable.

Third, more tree attributes can be estimated using the MSS method. The features examined in this study were the DBH and tree height, which are currently the most important tree attributes collected in forest inventories. The application of similar mapping techniques in the MSS method makes it possible for other tree attributes to be estimated on the sample plot. The applicability of the TLS data to collection of additional tree attributes has been reported for the leaf area index [25], volume [26], stem curve [27], understory biomass [28], vegetation density [29], and stem biomass [30]. Detailed models of foliage and branches may also be created from TLS data at the tree level [31]. By applying a mapping technique similar to that of the MSS method, the attributes of individual trees can be studied in the scan in which occlusion effects are minimal, and the stand attributes can be estimated by merging several individual estimations.

Last, the MSS method estimates the initial matching parameters of the point-level registration of several scans. The initial transform parameters of two point clouds consist of six parameters: three transitions in the XYZ directions and three rotations around the three axes. In practice, the scanner is kept level during the scanning. The initial estimation of two rotations around the X and Y axes can be zero. The transition in the Z direction can be estimated by the altimeter, which has begun to be integrated in scanners. Research is needed to determine the accuracy of the relevant height measurements and the accuracy required by the automated registration. The unknown initial estimations are the remaining three, two transitions in the XY plane and the rotation around the Z axis. The MSS method estimates all three of these unknown parameters.

The automated point-level registration generally comprises three steps, namely, the extraction of feature points, initial translation parameter estimation, and registration of the point clouds. Automated solutions for certain steps have been reported. With known stem points and initial translation parameters, registration using the cross-sectional centers of the stems was reported by Henning and Radtke [32]. The automated extraction of stem points and the cross-sectional centers of stems in dense forest plots using single-scan data were reported by Liang *et al.* [15]. The estimation of the initial transformation parameters is one last major technical challenges on this topic, which is discussed in this study. The road map of the automated co-registration of several TLS scans at the point level is now available by combining these three studies. The applicability of automatically matching TLS scans on forest plots at the point level should be tested.

The MSS method employs tree locations as the matching points. The features are the points that have their correspondences in the other data set. The rest of the points in the data set are noise. The challenge in this context involves the co-registration of data sets with considerable noise and local distortions.

The overlapping area covered by two scans varies depending on the scanning scenario and configuration. In this study, the point clouds employed had the same radius. The intersection area between the center and border scan is 68.5% of the area covered by the point cloud. Assuming that the trees were evenly distributed in the forest, the common trees between two scans are less than 70% of all trees captured by a point cloud.

Only a portion of the common trees can be used as feature points in the matching process. Some trees are not captured by the point cloud because of occlusion; others are not detected in the data interpretation. The accuracy of stem detection is strongly affected by the stem density, forest scene complexity, and measuring geometry [15]. It has been reported that 10–32% of trees were not available in the point cloud captured from the plot center because of the occlusion effect [12–15]. An additional 4–33% of trees available in the point cloud were not detected [13,14,24]. The detection accuracy in dense forests is clearly lower than in sparse ones, as shown in Table 4. Assuming that 20% of all the trees are not captured by the point cloud and 10% of available trees are not detected, the common trees between two scans account for approximately 50% of all trees in a scan. The exact percentage depends on the plot attributes, scanning configuration, and processing method. Therefore, in the matching problem addressed here, it is common that a tree in one scan does not have a corresponding one in another scan because its correspondence does not exist or is not detected in the point cloud.

Meanwhile, the detected stem locations may not match each other exactly because different parts of the same tree are captured by different scans. For example, the lower part of a leaning stem may be available in one scan while the upper part is present in another scan because of the scanning geometry. In such a case, the stem locations extracted from the two scans are distinct from each other, and thus local distortion is present in the data sets.

The MSS method employs a robust stem modeling procedure and a location-based matching technique to determine the transformations between scans. The modeling method is capable of reconstructing a stem model when undergrowth and lower branches are present. The location-based matching method is stable for noisy data sets, because it is not influenced by local distortions and individual errors. Therefore, it is capable of finding the registration parameters in data sets with considerable noise and local distortions.

## 6. Conclusions

This paper presents a fully automated solution for mapping stand attributes at the plot level using terrestrial laser scanning (TLS) and the multi-single-scan (MSS) method. The MSS method has elements of both the single-scan and multi-scan methods. This study demonstrates the possibility of merging individual scans at the feature and decision levels. The experimental results indicate that the MSS method achieves results for tree stem detection and DBH estimation that are similar to those previously reported for multi-scan methods, whereas artificial reference targets and point-level registration are not required in the proposed MSS method, leading to time savings in forest plot measurements. The results also shows that the MSS method improves the mapping results compared with those obtained using the single-scan approach. The MSS method can be used to estimate the initial registration parameters for the automated registration of several scans at the point level. Further research is needed to study the possibility of estimating other tree attributes at the plot level and to collect field reference for a large area using the proposed MSS method.

## Figures and Tables

**Table 1.** Descriptive statistics of the study plots.

**Plot**		**1**	**2**	**3**	**4**	**5**
Density (stems/ha)		605	732	764	764	1,210
Number (stems)		19	23	24	24	38
Species	Pine	94.7	0.0	70.8	87.5	0.0
(percent)	Spruce	0.0	73.9	4.2	8.3	97.4
	Deciduous	5.3	26.1	20.8	0.0	0.0
DBH	Min	7.4	6.6	5.7	11.7	5.7
(cm)	Max	23.0	34.2	29.6	24.7	40.9
	Mean	17.2	21.2	18.6	18.5	18.2
	std	4.0	8.5	6.8	3.3	8.7
Height	Min	12.0	4.4	5.9	6.0	5.5
(m)	Max	18.5	26.7	23.7	17.6	24.0
	Mean	16.4	19.1	16.9	14.4	17.1
	std	1.8	7.3	4.8	2.7	5.7

**Table 2.** Leica HDS6100 laser scanner specifications.

**Specifications**	**Leica HDS6100**
Field of view (°)	360 × 310
Measurement range (m)	79
Distance measurement accuracy at 25 m (mm)	±2
Data acquisition rate (points per sec)	508,000
Beam diameter at the exit (mm)	3
Beam divergence (mrad)	0.22
Max resolution (°)	0.009 × 0.009
Laser wavelength (nm)	650–690
Laser power (mW)	30
Weight (kg)	14
Operating temperature (°C)	–10–45

**Table 3.** The results of stem mapping using the MSS method.

**Plot**		**1**	**2**	**3**	**4**	**5**
Density (stems/ha)		605	732	764	764	1,210
Detection	(mapped stem/the total)	18/19	23/23	22/24	24/24	35/38
	(%)	94.7	100	91.7	100	92.1
DBH	Bias (cm)	0.69	0.23	0.70	0.11	0.63
	RMSE (cm)	0.90	1.64	1.90	0.97	1.79
	RMSE (%)	5.16	7.72	9.77	5.26	9.84
Height	Bias (m)	1.16	2.11	1.69	1.95	–0.34
	RMSE (m)	2.04	6.53	3.74	3.16	4.58
	RMSE (%)	12.47	34.11	21.21	22.02	26.34

**Table 4.** The results of stem mapping using the single-scan method.

**Plot**		**1**	**2**	**3**	**4**	**5**
Density (stems/ha)		605	732	764	764	1,210
Detection	(mapped stem/the total)	17/19	19/23	16/24	21/24	21/38
	(%)	89.5	82.6	66.7	87.5	55.3
DBH	Bias (cm)	0.76	0.50	–0.18	0.08	0.57
	RMSE (cm)	0.94	1.04	2.31	0.74	2.41
	RMSE (%)	5.32	4.69	10.34	3.94	12.37
Height	Bias (m)	1.12	1.26	0.54	1.50	–1.30
	RMSE (m)	2.02	4.29	1.36	2.25	3.81
	RMSE (%)	12.22	21.29	6.89	15.31	21.15

**Table 5.** The attributes of the stems that were not mapped (omissions) in the point clouds.

**Method**		**MSS**	**Single-scan**
Missed stems	(missed stem/the total)	6/128	34/128
	(%)	4.7	26.6
DBH	Min	5.70	5.70
(cm)	Max	32.70	34.20
	Mean	14.27	15.13
	std	9.94	8.47
Height	Min	8.00	4.40
(m)	Max	16.90	25.00
	Mean	11.13	13.95
	std	3.95	6.42
Distance *	Min	4.52	4.40
(m)	Max	8.18	9.79
	Mean	5.95	8.23
	std	1.33	1.28

* In the MSS method, the distance between the stem and the nearest scanner is measured. In the single-scan method, the distance between the stem and the center scanner is measured.

**Table 6.** Summary of the mapping results of the multi-scan method reported in references.

	**Plot**				**Results**	
	
	**Number**	**Size**	**Density (stems/ha)**	**Scan**	**Detection (%)**	**RMSE DBH (cm)**
Simonse *et al.* [6]	1	∼25 × 25 m	∼448	4	92.9	5.69 *
Thies and Spiecker [18]	1	∼30 × 30 m	555.6	5	52	–
Henning and Radtke [19]	1	20 × 40 m	–	15	–	8.9 **
Maas *et al.* [20]	1	12 m radius	309	3	100	1.48
Tansey *et al.* [21]	1	23 × 21 m	1031	4	– ***	1.9–3.7
Murphy *et al.* [14]****	18	30 × 33 m or 25 × 40 m	207–570	5	99.6	–
Huang *et al.* [22]	1	35 × 35 m	212	4	100	3.4–3.74

* RMSE is calculated from the mean error and standard deviation of the errors;

** Two trees with the smallest field-measured DBH were ignored;

*** 100% of the stems that could be identified manually. The overall mapping accuracy was not reported;

**** The processing of the TLS data was performed using commercial software. The method was reported in [23].
